# Improving Concrete Crack Segmentation Networks through CutMix Data Synthesis and Temporal Data Fusion

**DOI:** 10.3390/s23010504

**Published:** 2023-01-02

**Authors:** Maziar Jamshidi, Mamdouh El-Badry, Navid Nourian

**Affiliations:** Department of Civil Engineering, University of Calgary, Calgary, AB T2N 1N4, Canada

**Keywords:** crack segmentation, CutMix, data fusion, data synthesis, fully convolutional neural network, Transfer Learning

## Abstract

A key element in an automated visual inspection system for concrete structures is identifying the geometric properties of surface defects such as cracks. Fully convolutional neural networks (FCNs) have been demonstrated to be powerful tools for crack segmentation in inspection images. However, the performance of FCNs depends on the size of the dataset that they are trained with. In the absence of large datasets of labeled images for concrete crack segmentation, these networks may lose their excellent prediction accuracy when tested on a new target dataset with different image conditions. In this study, firstly, a Transfer Learning approach is developed to enable the networks better distinguish cracks from background pixels. A synthetic dataset is generated and utilized to fine-tune a U-Net that is pre-trained with a public dataset. In the proposed data synthesis approach, which is based on CutMix data augmentation, the crack images from the public dataset are combined with the background images of a potential target dataset. Secondly, since cracks propagate over time, for sequential images of concrete surfaces, a novel temporal data fusion technique is proposed. In this technique, the network’s predictions from multiple time steps are aggregated to improve the recall of predictions. It is shown that application of the proposed improvements has increased the F1-score and mIoU by 28.4% and 22.2%, respectively, which is a significant enhancement in performance of the segmentation network.

## 1. Introduction

Civil infrastructure systems, such as bridges, can be subjected over their service life to excessive loads and adverse environmental conditions. As a result, with the progress of time, cracks and other surface defects begin to appear. Therefore, routine inspection and maintenance are periodically required to ensure satisfactory serviceability and safe operation. Visual inspection of structures for surface defects is the conventional way that is widely adopted in practice. In concrete structures, cracks are considered an early sign of structural deterioration and, therefore, require careful monitoring. Geometric characteristics of concrete cracks such as their length, width, direction, and pattern of propagation are usually used to determine their severity. These are the parameters that are commonly estimated and recorded by an inspection crew during on-site investigations. However, performing on-site inspection can be labor-intensive, costly, error-prone, and unsafe, as well as disruptive to or interfering with the traffic. As a result, there is a surge of interest in automated inspection systems in which optical sensors are utilized to detect and locate visual defects and measure their properties using computer-based image processing techniques.

In recent years, there has been a surge of interest among researchers in utilizing machine learning techniques to predict visual surface defects, e.g., [1], as well as, mechanical strength of concrete structural components, e.g., [2]. With regard to crack detection, researchers have conventionally adopted different computer-vision algorithms to visualize crack locations in images. Edge detection techniques such as Canny and Sobel filters are traditionally used for crack detection [3]. However, these methods are sensitive to the image conditions such as camera distance, light conditions, and background [4]. Thus, the outcome of such techniques can be inconsistent. In search of more reliable techniques, recent studies focus on utilizing the power of deep convolutional neural networks (CNNs) for crack detection and segmentation. These studies demonstrated that fully convolutional neural networks (FCNs) have remarkable outcomes for crack segmentation which is the task of crack prediction at a pixel level [5,6,7,8,9,10]. The great advantage of FCNs is that they can process raw inspection images and produce high-resolution predicted crack masks. Effectively, when trained with large datasets FCNs can generate accurate predictions without pre-processing. However, the drawback of FCNs for concrete crack segmentation is that when trained with small datasets—such as those publicly available labeled images for crack segmentation—they may have poor performance when tested on images with different scenes and backgrounds. Thus, even though new architectures that are customized for various surface defection (e.g., SDDNet [11] and SrcNet [12]) are being developed by researchers, the practical application of segmentation networks may remain limited until more labeled datasets for crack segmentation become available. For the case of small training datasets, an effective solution to improve the accuracy and generalization of networks is to follow the method of Transfer Learning (TL). The concept of TL is to utilize the skills that a network learned when trained for a source task with a large dataset to improve the performance of the network for a target task with a small dataset. A common approach in TL is to reuse the parameters from a network pre-trained for the source task to initialize the parameters of the new network. The initialized network can then be fine-tuned for the target task using the relevant dataset.

In many applications, it is proved that when dealing with small datasets, CNNs trained using TL outperform those networks that are trained from scratch [13]. For surface damage detection it is common to employ networks pre-trained on general visual object detection datasets such as ImageNet [14]. For example, Gopalakrishnan et al. [15] used a popular CNN classifier called VGG-16 pre-trained on ImageNet to detect cracks on concrete and asphalt pavements. Gao and Mosalam [16] also utilized CNN classifiers pre-trained on ImageNet for structural damage classification. Zhang et al. [17] adopted a similar approach for pavement crack classification. Similarly, Dais et al. [18] adopted popular image classification networks, such as MobileNet [19], which were pre-trained on ImageNet for crack detection in masonry surfaces. In a similar approach, Yang et al. [20] fine-tuned the weights resulted from training on ImageNet for crack classification. For the task of road crack segmentation, Bang et al. [21] used ImageNet to pre-train the convolutional segment of an encoder-decoder network. Using a different dataset, Choi and Cha [11] employed a modified version of the semantic segmentation dataset Cityscapes [22] for pre-training a new crack segmentation network called SDDNet. Networks pre-trained on large-scale datasets such as ImageNet and Cityscapes with many different categories (e.g., cars, cats, chairs, etc.) have the skills necessary for detecting objects in a scene. However, these networks do not specifically learn the features associated with cracks. Additionally, these datasets may lack the common type of backgrounds that may appear in crack images.

A more efficient solution is to pre-train segmentation networks with the available crack datasets and use synthesized crack images for fine-tuning the network. The synthesized crack images should include the unique features that appear in an actual image. In a method called CutMix, a random patch from one image in the dataset is cropped and pasted onto another image to generate a dataset that resembles real conditions. This method was first introduced by Yun et al. [23] as a regularization strategy to improve generalization of CNN classifiers. They empirically demonstrated that CutMix data augmentation can significantly boost performance of the classifiers. However, randomly selecting the location of the cropped patches may result in a non-descriptive image and can limit its performance gain. Therefore, Walawalkar et al. [24] proposed an enhanced version of this method called Attentive CutMix where, instead of a random combination, only the most important regions of the image are cropped. These regions are selected based on the feature maps of another trained CNN classifier. Li et al. [25] incorporated Adaptive CutMix in their TL pipeline to expand the dataset of road defects. Following a similar mixing concept, in Mosaic data augmentation method four images are combined to form new training data. Yi et al. [26] used the Mosaic augmentation technique to increase the size of their training dataset for defect detection inside sewer systems. For the task of crack segmentation, however, the random selection of image patches may result in images that have no distinctive features associated with cracks. That is because cracks consist of thin linear shapes that occupy only a fraction of the image. Additionally, methods such as Attentive CutMix require an additional feature extractor network to pick up the regions that have the most relevant information which increases the complexity of the method. Therefore, in this study, a simple yet effective data synthesis method based on CutMix is proposed where the cropped patch is selected by considering the spread and distribution of cracks. This reduces the chance of generating non-descriptive crack images. Considering the significant discrepancy between the backgrounds of images in publicly available crack datasets and those of the images used in practice, in this study, in an automated manner, background information from uncracked scenes is employed to provide segmentation networks with a boost of performance. This can potentially reduce the possibility of false detection of background objects that resemble cracks, and thus, improve the precision of detection.

Another solution to improve accuracy, and hence performance, of existing segmentation networks is to incorporate post-processing algorithms. Particularly, for a sequence of crack images captured from the same structure at different points in time, the outcome of crack segmentation can be aggregated to improve the accuracy of the segmentation network. That is because cracks grow gradually, and their lengths and width increase with the progress of time. Therefore, detections from a future step can inform the position of cracks in the previous steps. Additionally, previous steps can be used to correct segmentation errors that might be caused by unexpected changes in the image conditions. Following this idea, in this study, a temporal data fusion method is proposed to enhance the outcome of segmentation networks. The proposed method is applicable for cases in which images of a concrete structure are sequentially captured over time. It is shown that the segmentation results of such images can be fused to improve the detection recall.

The remainder of this paper is organized as follows. Section 2 states the objective and significance of the research. Section 3 provides an overview of the proposed improvements. In Section 4, the details and training procedure of the segmentation networks are described. In Section 5, the proposed data synthesis technique is explained and validated. Section 6 describes details of the temporal data fusion and its effect on segmentation accuracy. Lastly, a summary and concluding remarks are given in Section 7.

## 2. Research Significance

The objective of this study is to increase utility of segmentation networks trained with public datasets when applied for continuous monitoring of concrete structures. Considering the limited number of publicly accessible images labeled for crack segmentation, it is likely that networks trained with those images be employed in settings with background scenes or surface conditions not represented in their training datasets. Reliability of these networks can, therefore, be limited by such mismatches to the point of rendering them useless. To address this issue, the contribution of the current study is twofold. Firstly, a technique for synthesizing labeled images is proposed in which background scene of the structure being monitored is incorporated to help the networks better distinguish cracks from background. Secondly, since in a continuous monitoring system visual information could be accessible over time, it is proposed to aggregate detection data in different time steps to further improve the accuracy of trained networks.

## 3. Framework Overview

**Figure 1:**
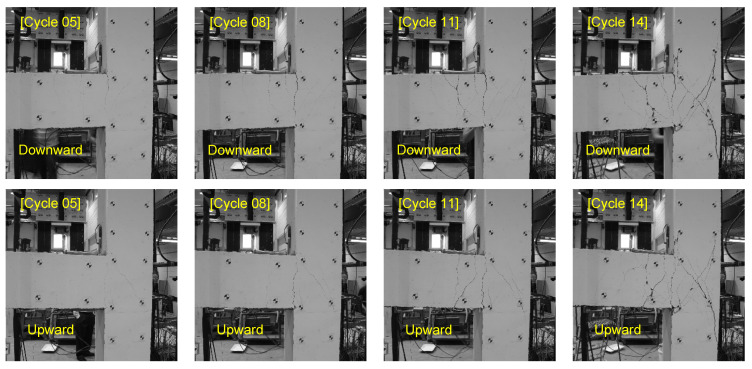
Examples of images of beam-column joint in a cyclic loading test used in a target dataset.

For training crack segmentation networks, researchers and practitioners should either produce their own dataset or rely on those that are publicly available. Manual labeling of crack pixels for crack segmentation is a time-consuming job. In fact, labeling crack pixels is more labor-intensive than annotation of images for classification and bounding box detection [27]. As a result, the amount of publicly available datasets for concrete crack segmentation is relatively small. The objective of this study is to enhance the performance of an FCN when trained on a small public crack dataset. To this end, the accuracy of the segmentation network is evaluated against a target dataset which is generated from a laboratory experiment of a concrete beam-column joint specimen [28]. The specimen underwent cyclic loading where the end of the beam is loaded in both upward and downward directions with an increasing amplitude until failure of the specimen. An image is captured at the peak or valley of each load cycle, therefore, all the cracks that developed during the experiment are included in the dataset. In total, 28 images were captured with an equal distribution between upward and downward loads. These images are utilized to create a target dataset to evaluate the trained network. Figure 1 illustrates examples of the captured images for both loading directions.

**Figure 2:**
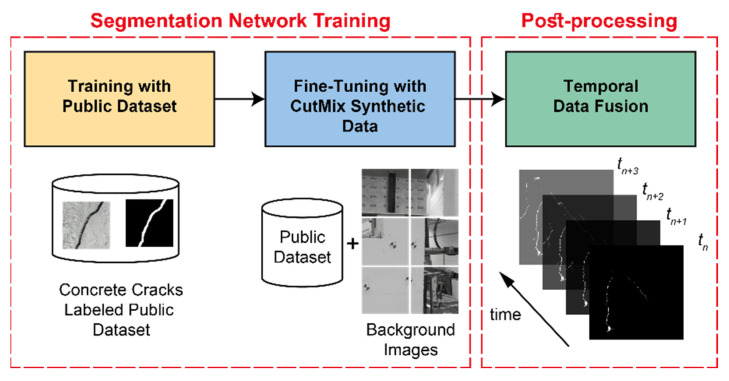
Overview of the proposed framework.

The improvements proposed in this study are outlined in Figure 2. In the proposed framework, the segmentation network trained on the public dataset is first fine-tuned using a dataset synthesized through the CutMix technique. In this technique, regions from the background images are mixed with the labeled public dataset in a systematic and automated manner. Thus, the network learns background information in the scene without losing its ability to detect cracks. Afterward, the crack masks predicted over time are combined following a temporal data fusion technique. Doing so takes advantage of the outcome of multiple frames to increase the accuracy of detected crack masks by retrieving those pixels that are misclassified as background.

## 4. Base Model

### 4.1. U-Net Architecture

**Figure 3:**
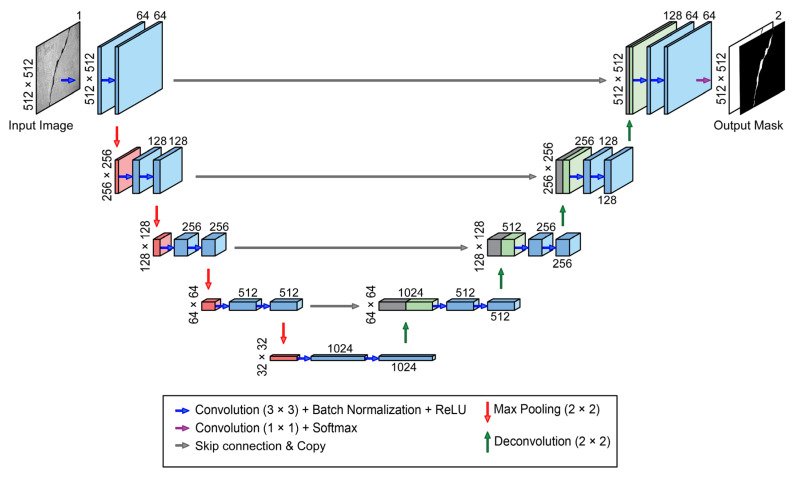
U-Net architecture.

U-Net is an end-to-end fully convolutional neural network proposed by Ronneberger et al. [29] for segmentation of biomedical imaging. Since its inception, U-Net was employed for the task of semantic segmentation in other applications including crack detection. In particular, Liu et al. [5] demonstrated that this network can accurately detect crack pixels in images from concrete surfaces. Here, U-Net is selected as a reference architecture to establish a base model. It should be noted that the improvements proposed in this study are independent of the choice of the segmentation network. Therefore, other newly developed architectures, such as U-Net++ [30], can also benefit from these improvements. The details of the U-Net architecture are shown in Figure 3. The contracting path of the network is responsible for feature extraction from the input image and the expanding path localizes crack pixels in the output mask. As shown in the figure, the input is a 512 × 512 grayscale image, and the output is a two-channel segmentation map for background and crack. The arrows represent the operations that result in the feature maps displayed as blocks. Convolutional operations shown with blue arrows have a kernel size of 3 × 3 with stride 1 and are followed by batch normalization and ReLU activation function. The purple arrow indicates convolutions of kernel size 1 × 1 which is followed by Softmax activation. Max-pooling down-sampling operations in the contracting path are shown with red arrows. Deconvolution up-sampling operations with a kernel size of 2 × 2 and stride 1 in the expanding path are indicated with green arrows. Gray arrows represent the skip paths where the feature maps from the contracting path are concatenated with their counterparts in the expanding path. The numbers above and below the blocks represent the number of channels.

### 4.2. Training and Cross-Validation on Public Dataset

**Figure 4:**
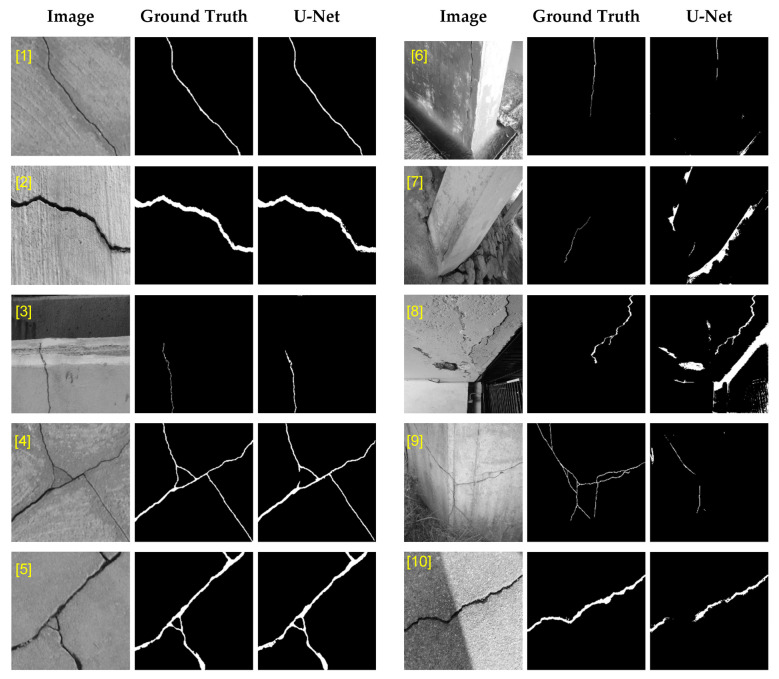
Samples of public dataset images and their corresponding masks ([1]–[5]: cases where U-Net successfully predicted the crack pixels; [6]–[10]: cases where U-Net missed parts of the crack or mistakenly detected other objects in the background as crack).

The described U-Net is trained and cross-validated using the labeled crack images extracted from three public datasets [31,32,33] and the dataset previously prepared by the authors based on the local bridge inspection images acquired from Hong Kong Highways Department [7,34]. In total, 540 crack images with their corresponding labels were collected. In Figure 4, samples of images in the evaluation dataset along with their corresponding mask predicted by U-Net and their ground truth are illustrated. The first five images are examples of the cases where U-Net could successfully predict the crack pixels; whereas images 6 to 10 show the cases where the network missed parts of the crack due to poor lighting, or mistakenly detected other objects in the background as crack. To prevent overfitting, a standard method is to perform data augmentation during training of the network. Data augmentation techniques improve generalization of the network by introducing random distortions that resemble actual imaging conditions. The augmentation techniques that are adopted in this study are horizontal and vertical flip, rotation (±0.2°), horizontal and vertical shift (±0.05), shear (±0.05°), and zoom (±0.05).

**Figure 5:**
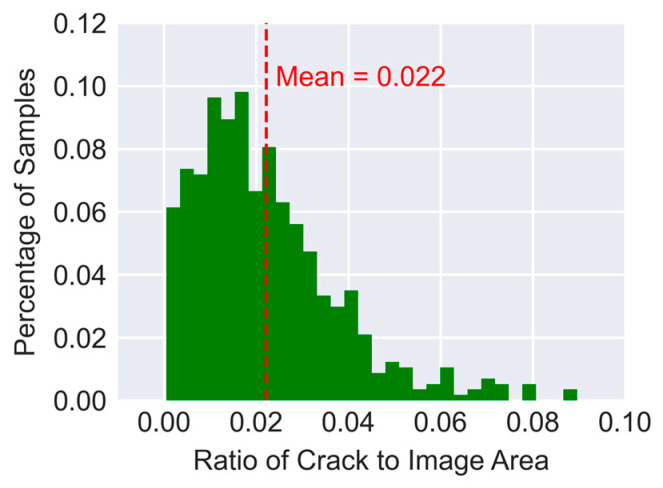
Distribution of crack-to-area ratio in the public dataset of 540 images.

A well-known problem related to the crack segmentation task is massive imbalance between the number of crack pixels and background pixels. Due to this imbalance, the ratio of the area occupied by cracks in an image to the total area of the image is often small. The histogram in Figure 5 demonstrates the distribution of crack-to-image area ratio for the entire public dataset. On average, only 2.2% of the area of an image in the dataset corresponds to crack. That is because cracks are thin linear shapes that include only a small fraction of the total number of pixels in an image. Consequently, for a typical cross-entropy loss function used for classification, the imbalance in the training data can significantly slow down training of the network. To address this problem Lin et al. [35] proposed Focal Loss (FL) which Equation (1) shows its representation for binary classification.

(1) $$\mathrm{Focal}\;\mathrm{Loss}=\{\begin{array}{cc} -\left(1-y{)}^{\gamma }\log(y\right) & \mathrm{if}\;\hat{y}=1\\ -y^{\gamma }\log(1-y) & \mathrm{if}\;\hat{y}=0 \end{array},$$

In this equation y and $\hat{y}$ are the predicted and ground-truth labels, respectively, and γ is the focusing parameter. In Focal Loss, for γ>0, the loss function is modulated in such a way that it down weights the error contribution of well-classified pixels while maintaining the contribution of misclassified pixels. In this way, the network focuses on learning hard crack pixels rather than easy background pixels. In this study, the focusing parameter γ is equal to 2.

**Figure 6:**
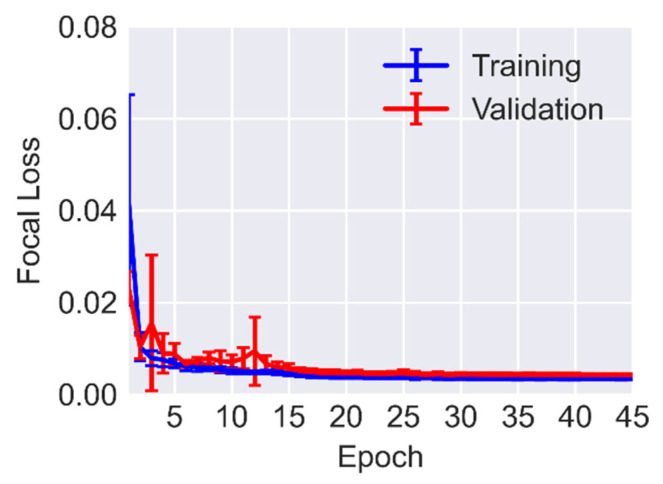
Focal Loss versus epochs for 5-fold cross-validation.

The network was trained with FL for 45 epochs with a mini-batch size equal to 2 images per step. The initial learning rate is 10^−4^ which was divided by 10 after every 15 epochs. The network parameters were optimized with Adam optimizer with $\beta_{1}=0.9$ and $\beta_{2}=0.999$. The implementation of U-Net was performed using TensorFlow and Keras libraries. To demonstrate the performance of the U-Net architecture on the training dataset 5-fold cross-validation was carried out. In this validation scheme, the dataset is randomly divided into 5 sections with equal sizes. For each fold of validation, one section is held out for validation and the rest is employed for training. In this way, the entire dataset has an equal chance to be used for both training and testing. Averaging the loss and accuracy metrics across all five possible combinations eliminates any bias that may result from data selection and thus provides a more reliable way for measuring the performance of the U-Net. Figure 6 displays the evolution of FL during training. In this figure, the blue and red lines show the mean values for training and validation datasets, respectively. The vertical lines display the variation of loss for all 5 folds of training and validation. The plateaued training and validation curves imply that the U-Net was trained for a sufficient number of epochs.

**Figure 7:**
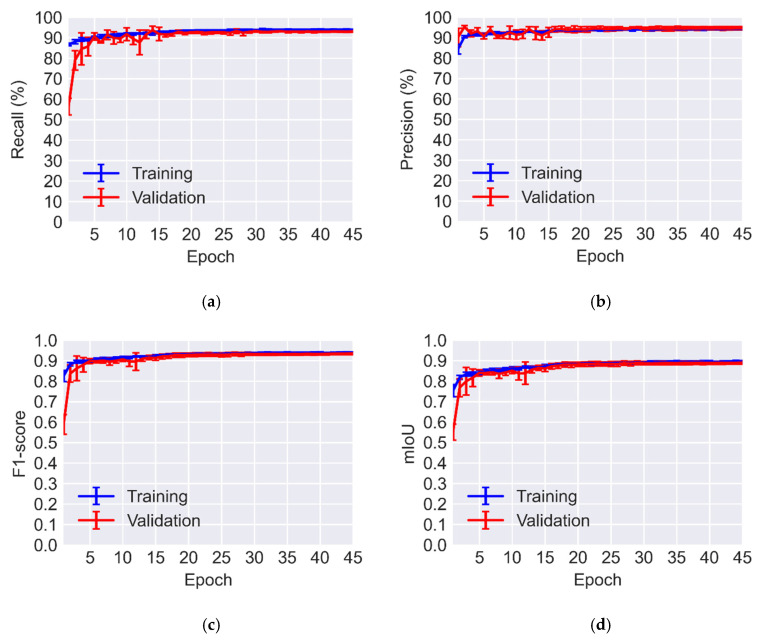
Accuracy metrics versus epochs for 5-fold cross-validation: (**a**) Recall; (**b**) Precision; (**c**) F1-score; (**d**) mIoU.

The variation of recall and precision are plotted in Figure 7. In addition to recall and precision, standard metrics to assess the accuracy of crack segmentation are F1-score and intersection over union (IoU). The mathematical representations of the accuracy metrics utilized in this study are as follows:

(2) $$\mathrm{Recall}\;=\frac{\mathrm{TP}}{\mathrm{TP}+\mathrm{FN}}$$

(3) $$\mathrm{Precision}\;=\frac{\mathrm{TP}}{\mathrm{TP}\;+\;\mathrm{FP}}$$

(4) $$F1-\mathrm{score}\;=2\frac{\mathrm{Precision}\times \mathrm{Recall}}{\mathrm{Precision}+\mathrm{Recall}}$$

(5) $$\mathrm{IoU}\;=\frac{\mathrm{TP}}{\mathrm{TP}\;+\;\mathrm{FP}\;+\;\mathrm{FN}}$$

In the above equations, TP, FP, and FN are the number of true positives, false positives, and false negatives, respectively. Another metric that can be utilized for binary classification tasks such as crack segmentation is Matthews correlation coefficient (MCC) which can be calculated by Equation (6), in which TN indicates the number of true negatives. MCC has a value between −1 and 1, where the latter represents perfect classification, and the former indicates complete disagreement between prediction and ground truth. Unlike other aforementioned metrics, MCC is a more reliable metric for imbalanced datasets such as crack images [36].

(6) $$\mathrm{MCC}\;=\frac{\mathrm{TP}\cdot \mathrm{TN}\;-\;\mathrm{FP}\cdot \mathrm{FN}}{\sqrt{\left(\mathrm{TP}\;+\;\mathrm{FP}\right)\cdot \left(\mathrm{TP}\;+\;\mathrm{FN}\right)\cdot \left(\mathrm{TN}\;+\;\mathrm{FP}\right)\cdot \left(\mathrm{TN}\;+\;\mathrm{FN}\right)}}$$

The validation recall and precision averaged for both classes of crack and background are 93.10% and 94.61%, respectively. The corresponding values for F1-score and mean IoU (mIoU) are 0.9346 and 0.8901. The average MCC across all folds is 0.8694. The obtained metrics reflect that the U-Net has a good performance when it is trained and validated on the public dataset. Moreover, these values are similar to those reported in other crack segmentation studies [5,11].

Receiver operating characteristic (ROC) curve is another measure that reflects how skillful the trained U-Net is in distinguishing crack pixels from background pixels. To obtain the ROC curve true positive and false positive rates are plotted for varying values of classification thresholds. The definition of true positive rate is the same as recall (Equation (2)) and false positive rate (FPR) can be calculated from Equation (7).

(7) $$\mathrm{FPR}\;=\frac{\mathrm{FP}}{\mathrm{FP}\;+\;\mathrm{TN}}$$

**Figure 8:**
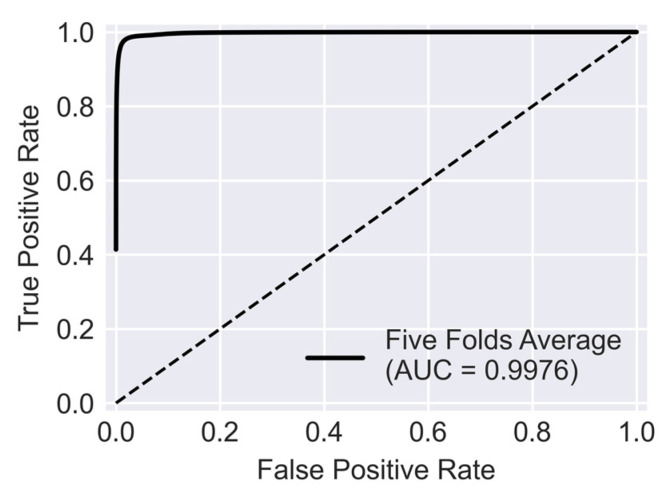
ROC curve for five folds of cross-validation.

Figure 8 illustrates the average ROC for five folds of cross-validation. The diagonal dashed line shows the performance of a hypothetical model where its classification skill is no better than random chance. Therefore, as a trained network becomes more skillful its ROC curve gets further away from this diagonal line and closer to the top left corner. The area under the ROC curve, also known as AUC, summarizes the performance of the network by a value between 0 and 1. AUC for the U-Net trained with the public dataset is 0.9976. This value implies that for the trained U-Net there is 99.76% chance to distinguish crack from background pixels which is an excellent performance.

### 4.3. Validation on Target Dataset

**Figure 9:**
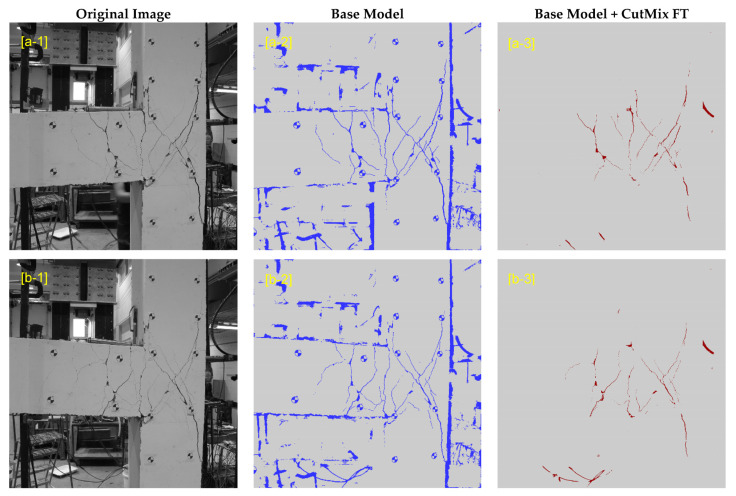
Samples of crack mask outcomes for the Base Model and the model fine-tuned with CutMix data synthesis for the last cycle of ([a-1]–[a-3]) downward and ([b-1]–[b-3]) upward loading (FT: fine-tuned).

**Figure 10:**
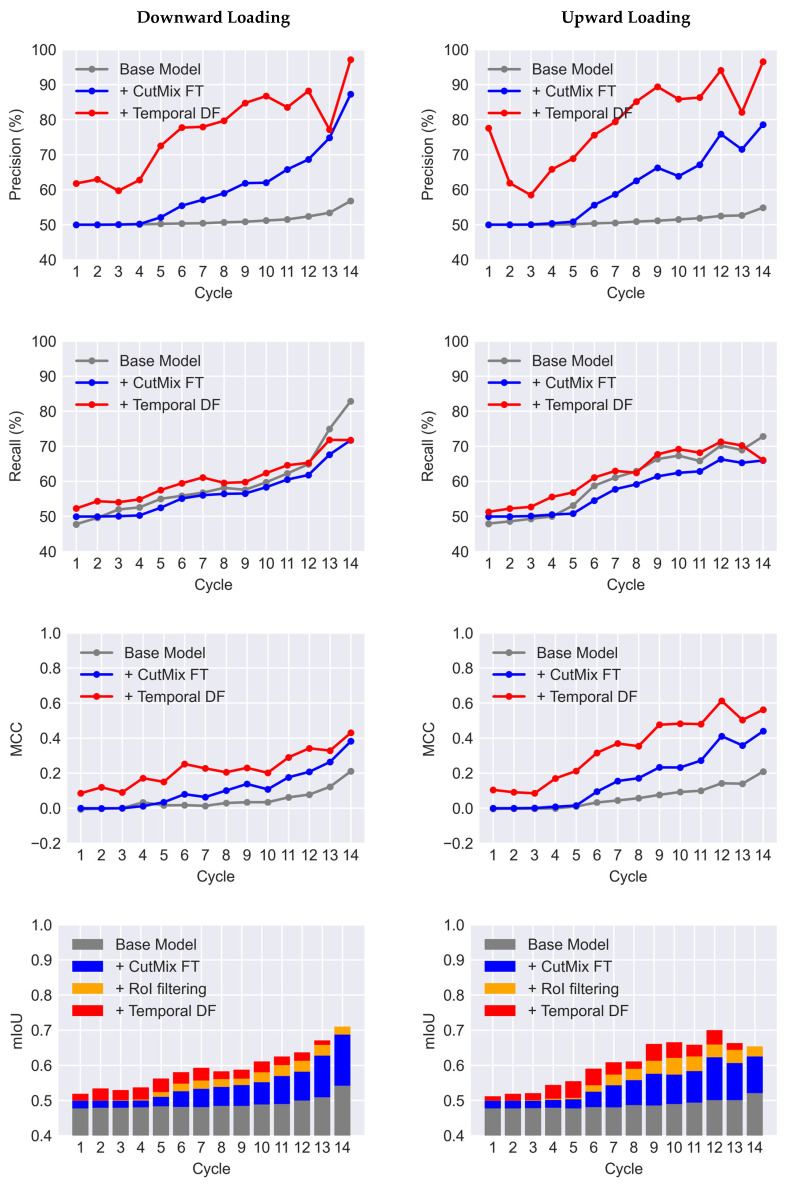
Breakdown of accuracy metrics for images of different cycles (FT: fine-tuning, DF: data fusion).

In this study, the goal is to employ the U-Net trained on the available public dataset to evaluate its performance when tested on a target dataset with different characteristics. Hereafter, the U-Net trained on the public dataset is called the “Base Model”. In the Base Model, all 540 images are used for training to take advantage of all available data. The original images captured from the concrete beam-column joint specimen used for evaluations have dimensions of 2592 × 2592 pixels. To create the target dataset, each image is divided into 81 number of 512 × 512-pixel tiles with a 252-pixel overlap in both horizontal and vertical directions. As a result, for 28 images, a set of 2268 images with 512 × 512 pixels were created. Every single tile can then be used as an input to the Base Model to obtain its corresponding crack mask. Subsequently, individual masks are stitched back together to create the crack mask of the entire image. The first column of Figure 9 shows the original images pertinent to the last cycle of upward and downward loading (i.e., Cycle 14). The outputs of the Base Model for these images are illustrated in the middle column. As can be seen, the Base Model can detect the actual crack pixels. However, it falsely detects objects in the background and circular markers on the specimen as cracks. The overwhelming number of these false positives decreases the precision of detection dramatically. In Table 1, the first row presents the mean values for the accuracy metrics of the Base Model when tested on all images in the target dataset. Compared to the case when the trained U-Net is tested on the public dataset there is a significant reduction in all metrics. These low accuracy values demonstrate the poor performance of the Base Model when it is used for concrete crack segmentation on a dataset different from what is trained with. In Figure 10, the variation of precision, recall, mIoU, and MCC are plotted for all 14 cycles of upward and downward loading. From the figure, it can be concluded that with the progress of the experiment the recall of detection improves significantly. This can be attributed to the fact that cracks in the later cycles have more distinct features. However, precision for all cycles remains low which is caused by false detections. Therefore, to boost the performance of the Base Model, the precision should be improved.

## 5. Fine-Tuning with Synthetic CutMix Data

As shown in Figure 9, the Base Model struggles with distinguishing actual cracks from background objects and surfaces. This is because the new scene of the target dataset is unique and had no representation in the training dataset. To address this problem, a solution is to fine-tune the model with new images with backgrounds similar to the target dataset. In accordance with the Transfer Learning procedure, the Base Model is used as a pre-trained model whose parameters are used to initialize the new model before fine-tuning. This helps the network to learn the features that are specific to the target dataset while maintaining its crack segmentation skills. In this study, during re-training of the U-Net, all layers are allowed to be updated and none of the parameters are frozen.

A solution for capturing background information is to use an image of a scene with no cracks. In the case of the beam-column joint specimen used in this study, an image before the beginning of the experiment can be utilized for this purpose. A major problem with re-training the U-Net with a dataset of no crack pixels is that it can make the network converge to a trivial solution where it assumes that the pixels consistently belong to the background. As a result, the U-Net can rapidly lose the skills learned to detect cracks. A more effective solution is combining the background information of the target dataset with the crack images in the public dataset. In this way, the network can be trained with a dataset that has crack information along with the features specific to the target dataset. The method proposed here to synthesize such a dataset is an extension to CutMix data augmentation technique proposed by Yun et al. [23]. The CutMix technique is intended to improve the generalization capability of the CNN classifiers. Here, it is repurposed to generate a dataset that is a mix of a public labeled dataset and the background information of the target dataset.

**Figure 11:**
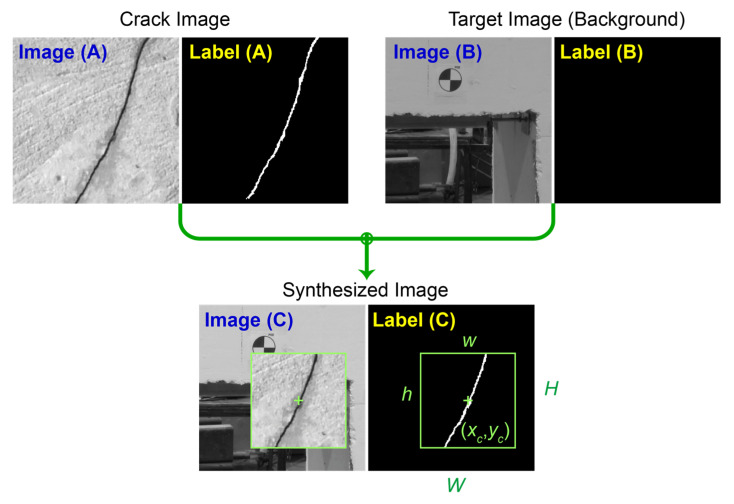
CutMix data synthesis procedure.

As illustrated in Figure 11, in this technique a pair of images are randomly selected. One image is a crack image, i.e., Image (A), drawn from the public dataset with its corresponding label. The other image, i.e., Image (B), is from a scene of the target structure when cracks are not initiated yet, therefore, its label, i.e., Label (B), contains no crack pixels. In this way, all pixels in Image (B) can be labeled as background. Here, both images have equal dimensions of 512 × 512 pixels. To combine these two images, a rectangular patch with the center at coordinates $\left(x_{c},y_{c}\right)$, displayed by the “+” symbol in Figure 11, and width, w, and height, h, is randomly generated. As shown in Figure 11, in the new combined Image (C), the area inside the rectangle is cropped from Image (A) and pasted in Image (B). Following a similar procedure, the corresponding Label (C) for the new image can be produced as well.

In the original CutMix technique, the coordinate for the center of the rectangular patch is drawn from a uniform distribution. For a general image classification task, where objects occupy a large portion of any given image, uniform random distribution for selecting the center point is a reasonable choice. However, for crack images, this approach can be problematic. The reason being since cracks are made up of small lines the odds of selecting a rectangle that includes no crack are high. As a result, the new dataset may have a significant number of samples with no crack information. To avoid this issue, a better approach is to select the center point from a multivariate normal distribution as shown in the following equation,

(8) $$\left(x_{c},y_{c}\right)\sim N\left(\mu ,\;\Sigma \right),$$

where the mean vector μ and the covariance matrix Σ are determined based on Label (A). These parameters can be calculated as in Equations (9) and (10). In these equations, $p_{i,j}\in \left\{0,\;1\right\}$ is the value of each pixel in Label (A) which is equal to 1 for crack pixels and zero for background pixels. Equation (9) calculates the mean vector which gives the coordinates of the center of the crack $\left(x_{o},y_{o}\right)$, and Equation (10) finds the covariance matrix which reflects the spread of the crack in both directions. The parameter α≤1 can be used to tighten the spread of the distribution. The lower α is the higher is the chance of positioning the center of the rectangle closer to the center of the crack.

(9) $$\mu ={\left[\begin{array}{cc} x_{o} & y_{o} \end{array}\right]}^{T}=\frac{1}{\sum_{j=1}^{H}\sum_{i=1}^{W}p_{i,j}}{\left[\begin{array}{cc} \overset{H}{\underset{j=1}{\sum }}\overset{W}{\underset{i=1}{\sum }}i\cdot p_{i,j} & \overset{H}{\underset{j=1}{\sum }}\overset{W}{\underset{i=1}{\sum }}j\cdot p_{i,j} \end{array}\right]}^{T},$$

(10) $$\Sigma =\frac{\alpha }{\sum_{j=1}^{H}\sum_{i=1}^{W}p_{i,j}}\left[\begin{array}{cc} \overset{H}{\underset{j=1}{\sum }}\overset{W}{\underset{i=1}{\sum }}{\left(i-x_{o}\right)}^{2}\cdot p_{i,j} & \overset{H}{\underset{j=1}{\sum }}\overset{W}{\underset{i=1}{\sum }}\left(i-x_{o}\right)\left(j-y_{o}\right)\cdot p_{i,j}\\ \overset{H}{\underset{j=1}{\sum }}\overset{W}{\underset{i=1}{\sum }}\left(i-x_{o}\right)\left(j-y_{o}\right)\cdot p_{i,j} & \overset{H}{\underset{j=1}{\sum }}\overset{W}{\underset{i=1}{\sum }}{\left(j-y_{o}\right)}^{2}\cdot p_{i,j} \end{array}\right].$$

As shown in Equation (11), the width and height of the rectangle are calculated through variable λ, where λ is sampled from a uniform distribution (Equation (12)). In this manner, the resulting rectangle maintains the aspect ratio of the underlying image.

(11) $$\begin{array}{cc} w=W\sqrt{\lambda }, & h \end{array}=H\sqrt{\lambda },$$

(12) $$\begin{array}{cc} \lambda \sim U\left(\lambda_{\min},\lambda_{\max}\right), & 0\leq \lambda_{\min}<\lambda_{\max}\leq 1 \end{array},$$

The lower and upper bounds $\lambda_{\;\min}$ and $\lambda_{\max}$ are introduced to control the ratio that the crack image is allowed to cover the background image. Lastly, for the cases where the generated rectangular patch goes beyond the image boundaries only the parts that are inside are considered and the outside region is cropped out.

**Figure 12:**
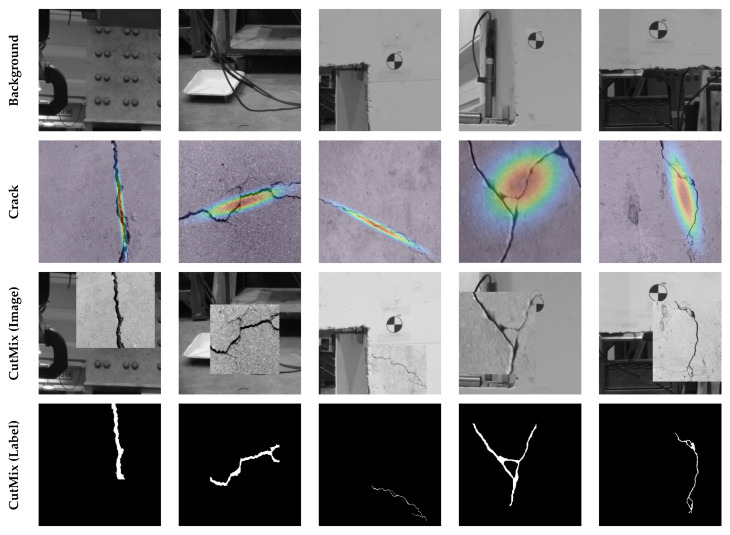
Samples of images and labels generated through the proposed CutMix data synthesis.

Figure 12 illustrates examples of images generated through the CutMix synthesis. The images in the first row are from the background of the target structure, and the images in the second row show examples of crack images from the public dataset. The contours overlayed on top of the crack images are the multivariate normal distribution derived from their corresponding binary mask. As shown, images with cracks propagated in multiple directions have wider distributions, and images with thin linear cracks have a narrow distribution in the major direction of the crack. These distributions determine the probability of the center points of rectangles used to create the CutMix images shown in the third row. In this study, it is assumed that α=0.5, $\lambda_{\min}=0.3$, and $\lambda_{\max}=0.9$.

The CutMix data synthesis is performed “on the fly” during fine-tuning of the Base Model. Following a similar tiling method described in Section 4.3, an image of the concrete specimen before the beginning of the experiment is selected and divided into 81 images. These background images are randomly mixed with a crack image from the public dataset of 540 images to generate new training samples. Meanwhile, the standard data augmentation techniques described in Section 4.2 are also performed on the crack images prior to CutMix synthesis. The Base Model was fine-tuned for 60 epochs with the learning rates of 10^−5^, 10^−6^, and 10^−7^ for the first, second, and third 20 epochs. Other hyperparameters remained unchanged during fine-tuning. In Figure 9, results of the fine-tuned model are demonstrated on samples of the target dataset. As can be seen, after fine-tuning with the CutMix synthetic data, the number of false positives is remarkably reduced. The network could effectively filter out background objects from the initial detections of the Base Model. As a result, the precision improves 8.94% (see Table 1). According to Figure 10, the improvement in precision becomes more pronounced for the later cycles of the experiment in such a way that for Cycle 14 of downward loading it jumps from 56.76% to 87.26%. The recall, however, has a slight 2% average reduction. Figure 10 confirms that for most cycles the recall decreases. Nonetheless, the F1-score, mIoU, and MCC have an increase of up to 0.17, 0.14, and 0.27, with an average gain of 0.07, 0.06, and 0.08, respectively.

**Figure 13:**
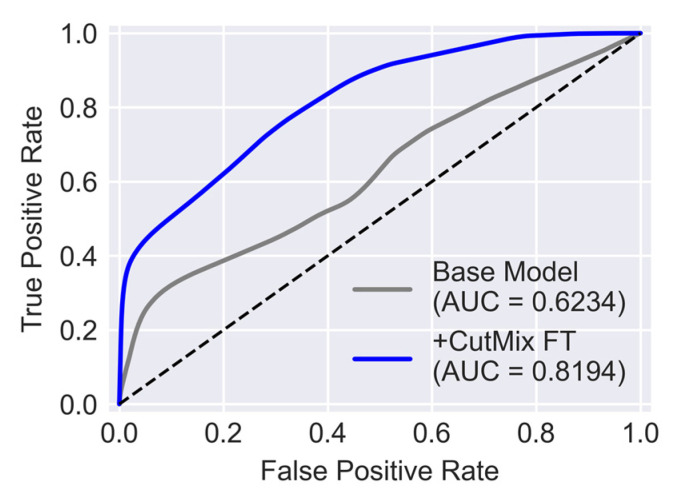
Comparison of ROC curves of models evaluated with target dataset.

In Figure 13, the ROC curve for the Base Model is compared with that of the model trained with CutMix synthesized data. Poor performance of the Base model when evaluated with the target dataset is evident by the fact that its ROC curve is closer to the diagonal line. Meanwhile, the model trained with CutMix data is relatively more skillful in separating crack pixels from background pixels. As a result, this model has an AUC equal to 0.8194 which, compared to the AUC of the Base Model equal to 0.6234, is a significant improvement.

## 6. Temporal Data Fusion

**Figure 14:**
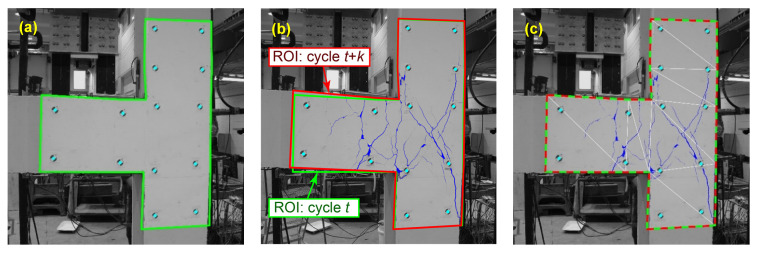
Process of mapping crack masks from cycle *t + k* onto cycle *t*: (**a**) image of cycle *t* with its ROI shown in green, (**b**) image and crack mask of cycle *t* + *k* with its ROI shown in red, and (**c**) transformed crack mask of cycle *t* + *k* overlayed on the image of cycle *t*.

Segmentation networks can easily miss thin cracks which results in a reduction in their recall. However, when a set of images from various cycles of a developing crack is available, detected thick cracks in future steps can be employed to locate thin cracks in the past steps. That is because crack propagation is a gradual process and thin cracks in images of earlier cycles turn into larger and more distinct cracks later. Thus, by fusing the data of multiple steps in the sequence of images crack pixels that are initially detected as background can be retrieved. The first step to aggregate the segmentation outcomes of multiple cycles is to correct the relative movements due to the structural deformations. Doing so requires tracking unique features in the images. In this study, as can be seen in Figure 1, there are 14 fiducial circular markers attached to the surface of the concrete specimen [37]. These markers can be utilized to track feature points in various images. The precise center point of each marker is found through the Normalized Cross-Correlation (NCC) method [38]. Based on the center points of these markers a region of interest (ROI) can be defined similar to the green polygon shown in Figure 14a. In the defined ROI, each corner of the polygon has a fixed distance relative to the closest marker’s center point. Given that the position of the camera remained unchanged for all images, the center points can be used as key points to make a transformation between ROIs in different cycles and thus cancel the effect of structural deformations. Figure 14b illustrates the image and crack mask of cycle *t + k*. In this image, the ROIs corresponding to steps *t* and *t + k* are drawn in green and red, respectively. By matching the key points of these two ROIs, a transformation can be created that maps the crack mask from cycle *t + k* back onto cycle *t*. Since the concrete specimen underwent a non-rigid deformation, a piecewise linear transformation was used in this study. The ROI of cycle *t + k* is divided into 12 triangles and the key points of each triangle are matched with the corresponding points in step *t* via an affine transformation. Figure 14c displays the result of the transformed mask overlaid on the image of step *t*. The affine transformation is done using MATLAB Image Processing Toolbox.

**Figure 15:**
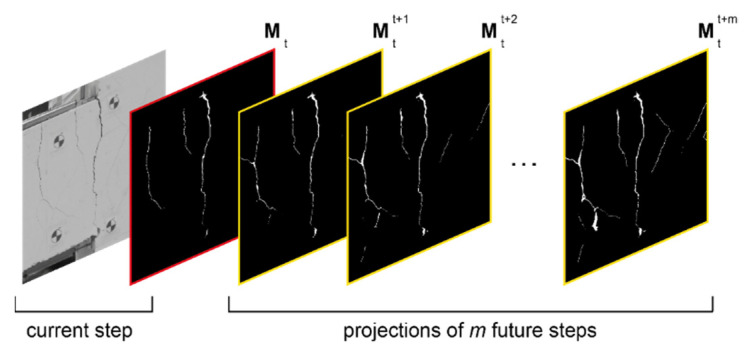
A set of crack masks corresponding to cycle *t* used for temporal data fusion.

Following the described mapping process, as shown in Figure 15, for cycle *t* a new projected crack mask $M_{t}^{t+k}\in {\left\{0,\;1\right\}}^{W\times H}$ can be defined as the crack mask from cycle k ahead mapped onto the ROI of the current step *t*. In this figure, $M_{t}\in {\left\{0,\;1\right\}}^{W\times H}$ is the crack mask of the current step. Therefore, using Equation (13) the effect of m future steps can be accumulated by defining $M_{t}^{*}\in {\left[0,\;1\right]}^{W\times H}$ as the weighted average of all future predictions.

(13) $$M_{t}^{*}=\sum_{k=1}^{m}w_{k}M_{t}^{t+k},$$

In this equation, $w_{k}$ is the weight associated with each considered step. Since closer steps have higher correlations with the current step it is reasonable that they receive higher weights. Thus, following a linear decay rule, the weight of step *k* can be calculated as in Equation (14).

(14) $$\begin{array}{cc} w_{k}=\frac{2m+2-2k}{m\left(m+1\right)}, & k=1,\;2,\ldots ,m \end{array},$$

The purpose of the proposed data fusion is to capture the crack pixels that are falsely detected as background. Therefore, the future steps should only affect the background pixels. Equation (15) is the mathematical representation of this constraint. In this equation, 1 is a matrix of ones with dimensions *W* × *H*, and operation “⊙” is element-wise multiplication. Additionally, output of the indicator function 𝕀(.) is equal to one for elements of $M_{t}^{*}$ that are greater than 0.5, otherwise, it is equal to zero. Lastly, ${\tilde{M}}_{t}$ is the final crack mask resulting from the proposed temporal data fusion procedure.

(15) $${\tilde{M}}_{t}=M_{t}+\left(1-M_{t}\right)⊙I\left(M_{t}^{*}\geq 0.5\right),$$

In the current study, all the future cycles are included to find the weighted average $M_{t}^{*}$ (Equation (15)). Additionally, only images of loading directions that are consistent with the current step are considered in data fusion. That is because the propagation of cracks in concrete elements depends on the direction of loading. As a result, during the upward and downward loading the geometry of crack patterns is different. As shown in Table 1, application of the described data fusion method on the target dataset has a significant effect on the accuracy of the resulting crack mask. The average precision and recall have a 17.28% and a 4.03% increase, respectively. Additionally, the average F1-score and mIoU improved to 0.6564 and 0.5975, respectively. It should be mentioned that the increase in the precision can be attributed to the fact that during the data fusion process false detections outside the defined ROI are filtered out. However, the increase in recall is directly due to the improvements achieved through the application of data fusion. Specifically, data fusion could capture false negatives using the information from the future steps. As shown in Figure 10, data fusion has positive impacts on the recall of all cycles from 1 to 13. Cycle 14, as the final step in the sequence, could not benefit from data fusion, thus, for this cycle all metrics remained unchanged. To better evaluate the performance of the proposed data fusion method in the plot of mIoU the increase due to ROI filtering is separated from the improvements resulting from data fusion. As shown, especially for the earlier cycles, data fusion has a significant impact which can be up to 8.6% relative improvement.

**Figure 16:**
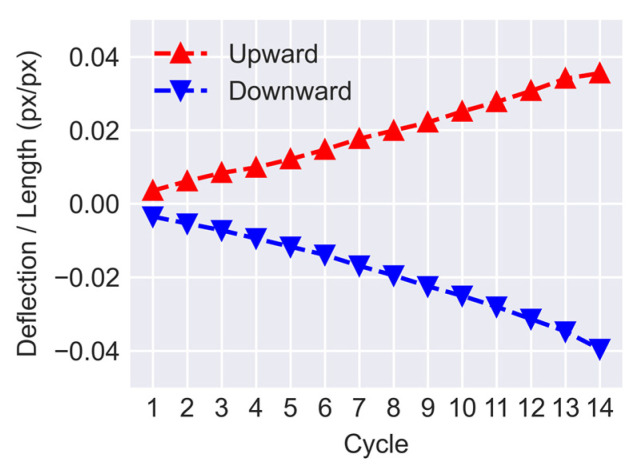
Deflection ratio of the concrete specimen.

The weight defined in Equation (14) puts more emphasis on the closer steps and discounts the effect of further steps. In this study, the weight of each step is assigned based on a linear decay rule due to its simplicity. However, it should be noted that the only hyperparameter that needs to be selected is the total number of steps considered for data fusion which depends on the rate at which cracks are growing between cycles. Figure 16 displays the ratio of the beam’s deflection over its length for cycles 1 to 14. The displacements and lengths are measured in the pixel domain using the position of the circular markers attached to the specimen. As can be seen, for both downward and upward loadings the deflection ratio of the beam is approximately changing linearly over time with an average difference of 0.003 between each consecutive cycle. A large interval between cycles can affect the outcome of crack mask transformations. Typically transformed masks of further steps have more misalignment errors when compared with the current step. That is due to larger deformations of the structural elements and the relative out-of-plane motion of the markers. The effect of such errors can be reduced by either using shorter intervals or more markers which can result in a finer mesh and a more accurate transformation.

Lastly, it should be noted that the proposed framework is able to approximately detect crack widths as small as 2.0 pixels. Based on the parameters of the camera and the configuration of the setup, the minimum crack width that the proposed framework is capable of detecting is around 1.0 mm. The ability of the proposed framework to detect thinner cracks can be enhanced by using a camera with a longer focal length or by capturing images at a shorter distance.

## 7. Summary and Conclusions

This study focuses on enhancing the accuracy of segmentation networks for concrete crack detection. Considering the small size of publicly available datasets for concrete crack segmentation, it is demonstrated that direct application of trained networks on a new dataset with different characteristics can result in poor performance. U-Net architecture, as a popular segmentation network, was trained on a public dataset to establish a Base Model. It is illustrated that such a model results in low precision when tested on a target dataset. The target dataset employed in this study is based on the images obtained during a cyclic loading test of a concrete beam-column joint specimen in a laboratory setting. The low accuracy of the Base Model is mainly due to the overwhelming number of false positives. Therefore, to improve precision of the network, the Base Model is fine-tuned with a synthetic dataset generated using a proposed CutMix method. In the proposed method, background images from the target structure are combined with the crack images from the public dataset. Meanwhile, during the combination process, the distributions of crack masks are taken into. As a result, only the most relevant region of the crack image is mixed with the background to avoid producing non-descriptive synthetic crack images. By fine-tuning the Base Model using the synthetic data it is shown that the average precision increases from 51.21% to 60.53%, which is a significant improvement. Therefore, it can be concluded that using the proposed method the segmentation network can learn the unique background features of a target dataset while maintaining its crack segmentation ability. It should be mentioned that the proposed data synthesis technique is based on the assumption that images of the intact states of structures are available. This is a valid assumption in long-term monitoring systems where histories of structures’ visual information are recorded. However, it may be a limitation in circumstances when images of structures’ initial states cannot be acquired.

Moreover, in this study, a temporal data fusion method is developed for the case where a sequence of crack images from the same concrete structure is available. The proposed data fusion method is a post-processing approach that leverages the shared information among multiple images to aggregate the detection outcomes from other time steps. Doing so improves the accuracy of detection by retrieving the pixels that are falsely detected as background. It is shown that for the target dataset used in this study this method can improve the recall from 57.24% to 61.27%. It should be noted that in this study deformations of the specimen components, i.e., the beam and the column, are corrected by using makers attached to the specimen. However, for more practical situations with no designated markers feature detection algorithms, such as scale-invariant feature transform (SIFT) [39], can be employed to find the key points on the structure. Overall, it is illustrated that the proposed techniques can improve the F1-score and mIoU of the crack segmentation of U-Net from 0.5113 and 0.4888 to 0.6564 and 0.5975, i.e., by 28.4% and 22.2%, respectively. Meanwhile, MCC increases from 0.0541 to 0.2831, which is a remarkable improvement for this accuracy metric. The achieved accuracy metrics are still below the case when U-Net is tested with a dataset similar to its training dataset. However, applying the proposed improvements can boost the performance of the network significantly.

The methods proposed in this study can be applied for long-term continuous inspection or laboratory testing of concrete structures. Here, images from a fixed camera are used to validate the methods. However, these methods are also applicable to cases where images are captured by a non-stationary camera as long as the camera’s path is known or can be retrieved accurately. Furthermore, an area of future study involves application of the proposed techniques to target datasets composed of images captured in the field.

## Figures and Tables

**Table 1:** Accuracy of models when tested against the target dataset (FT: fine-tuned).

Model	Precision (%)	Recall (%)	F1-Score	mIoU	MCC
Base	51.21	59.71	0.5113	0.4888	0.0541
Base + CutMix FT	60.53	57.24	0.5852	0.5492	0.1406
Base + CutMix FT + Data Fusion	77.81	61.27	0.6564	0.5975	0.2831

## Data Availability

The data derived from nonpublic domains are available from the corresponding author M.E.-B., upon request.
